# Clinical characteristics of familial and sporadic inflammatory bowel disease in Egyptian patients

**DOI:** 10.1186/s12876-025-04492-9

**Published:** 2025-12-15

**Authors:** Abobakr K. A. Talha, Sara Abdelhakam, Ahmed Nagah, Marwa Rushdy, Marwa A. Karim, Nada H. Abdel-Fattah, Shimaa Y. Kamel, Mohamed Eltabbakh

**Affiliations:** 1 Department of Tropical Medicine Faculty of Medicine, Ain Shams University, Cairo, 11566 Egypt; 2 Department of Clinical Pathology Faculty of Medicine, Ain Shams University, Cairo, 11566 Egypt; 3 Department of Ophthalmology Faculty of Medicine, Ain Shams University, Cairo, 11566 Egypt; 4 Department of Medical and Clinical Genetics Faculty of Medicine, Ain Shams University, Cairo, 11566 Egypt

**Keywords:** Inflammatory bowel disease, Familial, Sporadic, Egypt, Treatment response, Predictors, Mucosal healing.

## Abstract

**Background:**

Familial aggregation of inflammatory bowel disease (IBD) has been observed, but data from the Middle East, particularly Egypt, remain limited. This study investigated clinical differences, treatment responses, and predictors of response in familial and sporadic IBD patients.

**Methods:**

This case‒control study was conducted on ninety IBD patients (30 familial, 60 sporadic). Clinical history, inflammatory markers, endoscopic findings, and treatment outcomes were analysed over a one-year period. Predictors of response and mucosal healing were assessed via logistic regression.

**Results:**

Mucosal healing was achieved in a greater percentage of patients in the familial group (73.3%) compared to those in the sporadic group (55%), but the difference did not reach statistical significance (*P* = 0.093). The percentages of patients with mucosal healing, clinical response, clinical remission and normalization of C-reactive protein (CRP) levels at one year were significantly greater in ulcerative patients in the familial group [17 (85%), 18 (90%), 18 (90%) and 18 (90%), respectively] than in ulcerative patients in the sporadic group [21 (53.8%), 19 (48.7%), 19 (48.7%) and 20 (51.3%), respectively], with p values of 0.018, 0.002, 0.002 and 0.003, respectively. However, there was no statistically significant difference between Crohn’s disease patients of both groups regarding the outcome of the studied patients. A lower platelet count (≤ 324 × 10³/µL) and faecal calprotectin concentration (≤ 679 µg/g) were significant predictors of mucosal healing in familial cases (*P* = 0.012, *P* = 0.008, respectively).

**Conclusion:**

Familial IBD patients may present with a more severe initial inflammatory response but have better long-term treatment outcomes. These findings suggest that genetic and environmental influences play a role in IBD, highlighting the need for region-specific studies.

**Supplementary Information:**

The online version contains supplementary material available at 10.1186/s12876-025-04492-9.

## Background

Inflammatory bowel diseases are a spectrum of chronic idiopathic autoimmune inflammatory disorders with remission and relapses, primarily affecting the gastrointestinal system. There are two types, namely, ulcerative colitis (UC) and Crohn’s disease (CD) [1].

The prevalence of IBD has increased in many regions of the world, resulting in a substantial social and economic burden on healthcare systems. IBDs occur at varying frequencies worldwide. The countries reporting the highest incidence of UC are the United States, the United Kingdom and Sweden. IBDs have always been rare in the Middle East and North Africa. However, no accurate registry of patients has been established to study the exact prevalence of CD and UC in these populations. The largest cohort of Egyptian patients with IBD included 1104 patients with an established diagnosis of IBD between 2018 and 2021 [2].

Although genetic, environmental, and immunologic factors are involved in the pathogenesis of IBD, the aetiology of IBD is still not completely understood. Some suggest that a family history of IBD may be one of the most critical risk factors [3]. Other factors associated with this disease include nonsteroidal anti-inflammatory drug (NSAID) use, psychological stress factors, smoking history, and the consumption of milk products. Certain types of food compositions and the use of oral contraceptives may be associated with this condition. Some evidence indicates that smoking may be protective against ulcerative colitis, but a causal association remains unclear [4].

A family history of IBD was shown to increase the risk of developing IBD 10- to 15-fold in unaffected first-degree relatives and threefold among close relatives of IBD patients [5–7]. Even if genetic factors are associated with familial IBD [8], a family history of IBD does not mean that all patients with IBD share a specific gene, as family members of IBD patients could be exposed to similar environmental factors [9]. Some studies have shown that there are no differences in clinical characteristics between familial and sporadic IBD [10, 11]. Even if other studies demonstrated differences between familial and sporadic IBD, there have not been consistent results [12, 13].

Despite several studies on familial IBD, there is still insufficient knowledge regarding the distinct characteristics of familial and sporadic IBD in the Middle East region and North Africa, including Egypt.

This study aimed to investigate the differences in the clinical characteristics and disease course between familial and sporadic IBD patients in our country.

## Methods

**Fig. 1:**
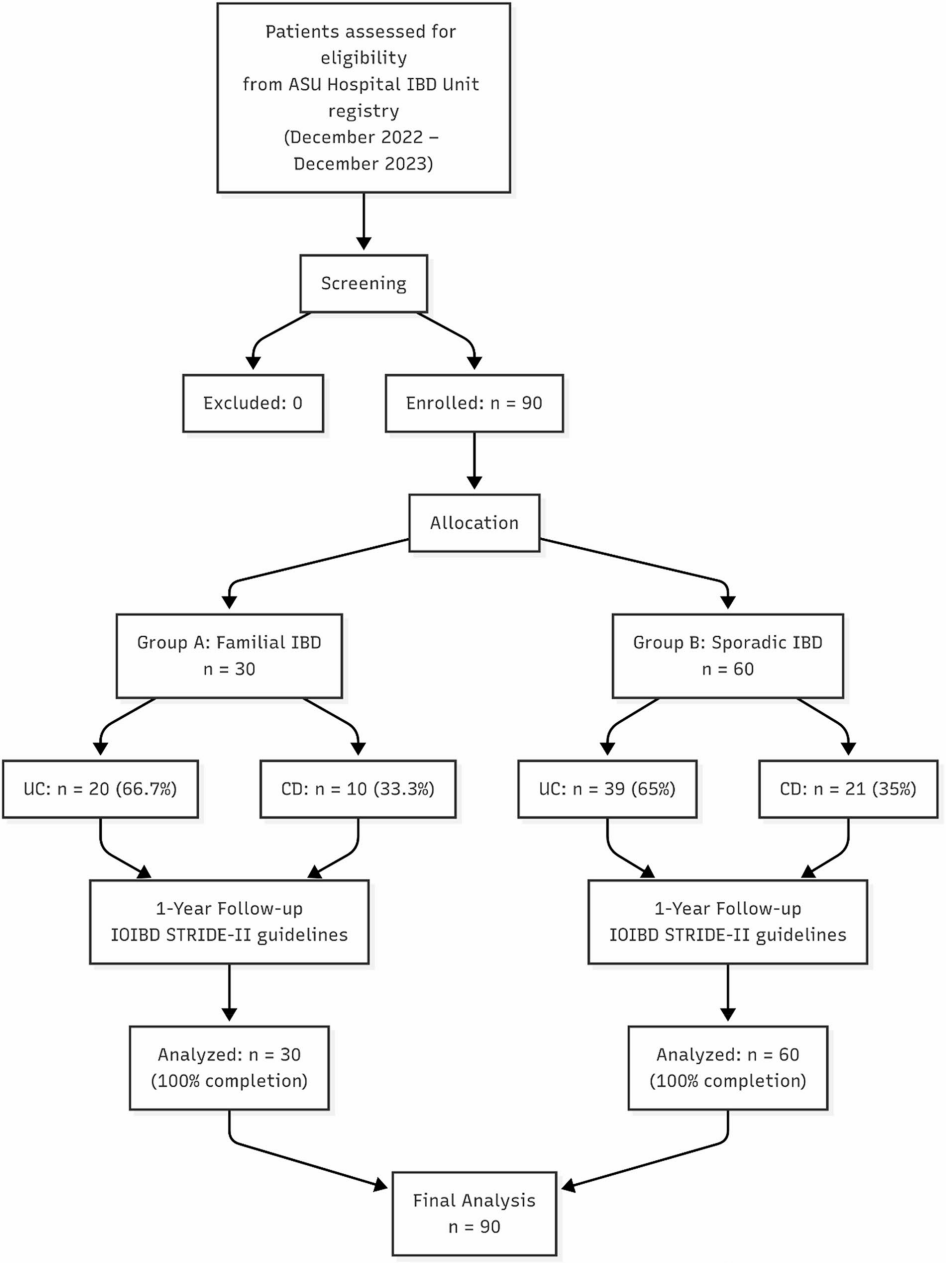
CONSORT flow diagram showing patient selection and allocation

This study is a case-control study. A case is defined as any IBD patient with a familial history of IBD, including first- and second-degree relatives. The control group was defined as any IBD patient without a family history of IBD who was matched for age, sex, and diagnosis (two times the number of cases). An a priori sample size calculation was performed using G*Power software. Since there are no population-based effect-size estimates from familial compared with sporadic IBD outcomes that are specific to Egypt, we chose to use the effect-size measure reported by Chung SH et al. (2014), as this is the most similar study that has been published. Our choice is well-supported by country-level and regional evidence, modelled GBD estimates indicate that Egypt’s age-standardised IBD prevalence increased from ~ 18 per 100,000 in 1990 to ~ 27 per 100,000 by 2017, and Arab-region report modelled prevalence rising to ~ 29–34 per 100,000 by 2019 [14]. Also, there has been an increase in the low prevalence in more recently reported data from Egyptian centres [2]. Korean estimates are higher than Egyptian values, ~ 100–200 per 100,000 [15], but remain much lower than in Europe and the United States ~ 800 per 100,000 [14, 16, 17]. As a result, using the Korean effect size is a conservative choice for our Egyptian study. With power set at 80%, alpha error at 5%, effect size of 0.4, and a case-to-control ratio of 1:2, the calculation determined that a sample size of 75 participants (25 familial IBD and 50 sporadic IBD) would be sufficient to detect differences between groups. Our final study population of 90 patients (30 familial and 60 sporadic) exceeded this requirement. The study was conducted from December 2022 until December 2023. The path of participants during study, covering the steps of screening, evaluation of eligibility, and the final designation into familial and sporadic groups, has been depicted in Fig. 1, no patients were excluded from the study, as all the eligible participants completed the entire one-year follow-up period without any issue. We included all patients diagnosed with inflammatory bowel disease, as well as their first- and second-degree relatives who presented to our Inflammatory Bowel Disease (IBD) Unit, which was established in 2009, within the Tropical Medicine Department at Ain Shams University Hospital, one of the largest tertiary hospitals in Egypt. Additionally, we included patients with confirmed inflammatory bowel disease who did not have a family history as controls. All patients were 18 years of age or older. We included all eligible patients in our study with a one-year follow-up. Our primary objective was to compare familial with sporadic cases. Comparing the subgroups of UC and CD is considered a secondary result.

All patients (cases and controls) were subjected to full history taking and complete clinical examination with special emphasis on abdominal pain, weight loss, rectal bleeding, diarrhoea, constipation, malaise, lethargy, anorexia, nausea, tenesmus, abdominal distension, mucous passage, vomiting, low-grade fever, environmental factors (such as residence, smoking, diet, contact with animals, occupation, travel history, infection, temperature and contamination), drug history and history of appendectomy or other operations. Baseline clinical data were collected from clinical records and patient interviews. Intolerance to milk products based on patient self-reporting of symptoms (e.g., bloating, diarrhoea) following the ingestion of dairy products; no formal test was routinely performed. A fully detailed family history for affected cases was taken, with three generations of family pedigrees drawn to all cases, laboratory investigations (at diagnosis and at the one-year follow-up), including CBC (complete blood count), serum creatinine, blood urea nitrogen, serum albumin, AST (aspartate aminotransferase), ALT (alanine aminotransferase), serum bilirubin, ESR (erythrocyte sedimentation rate), CRP titre and fecal calprotectin, and abdominal ultrasound), to exclude the presence of associated diseases or complications, and colonoscopy and biopsy were performed (at diagnosis and one-year follow-up). To confirm the diagnosis of IBD, to differentiate UC from Crohn’s disease and to assess the disease activity and degree of inflammation, the Mayo score and Truelove criteria for ulcerative colitis and CDAI (Crohn’s Disease Activity Index) for Crohn’s disease at diagnosis were calculated at the time of initial diagnosis at the hospital and after one year of follow-up.

Each patient in the two groups (familial or sporadic) was followed up for one year from the time of diagnosis to detect the response to treatment (clinical response, clinical remission and mucosal healing) according to the International Organisation for the Study of IBD (IOIBD) guidelines for Determining Therapeutic Goals for Treat-to-Target strategies in IBD [18]. Extraintestinal manifestations, including those affecting the musculoskeletal and mucocutaneous systems, the ocular tract, and the hepatobiliary tract, have been reported in these patients. Eye assessment (by a specialised ophthalmologist), which included assessment of visual acuity, anterior segment examination via slit lamp biomicroscopy, fundus examination and intraocular pressure measurement, was reported. The clinical characteristics and disease courses of familial CD patients, UC patients, and sporadic CD patients were compared. The responses to several types of treatment modalities were also reported for the two groups.

Data were collected, revised, coded and entered to the Statistical Package for Social Science (IBM SPSS) (IBM Corp. Released 2020. IBM SPSS Statistics for Windows, Version 27.0. Armonk, NY: IBM Corp). The quantitative data were presented as mean, standard deviations and ranges when parametric and median, inter-quartile range (IQR) when data found non-parametric. Also, qualitative variables were presented as number and percentages. The comparison between groups regarding qualitative data was done by using *Chi-square test* and/or *Fisher exact *test when the expected count in any cell found less than 5. The comparison between two independent groups with quantitative data and parametric distribution was done by using *independent t-test *while with non-parametric data were done by using *Mann-Whitney test*. Also, the comparison between more than two groups with quantitative data and parametric distribution was done by using* One Way ANOVA test* while with non-parametric data were done by using *Kruskall-Wallis test. Logistic regression analysis *was used to assess the odds ratio (OR) with their 95% confidence interval (CI) for predictors of mucosal healing and response. The confidence interval was set to 95% and the margin of error accepted was set to 5%. So, the p-value was considered significant at the level of < 0.05.

## Results

**Fig. 2:**
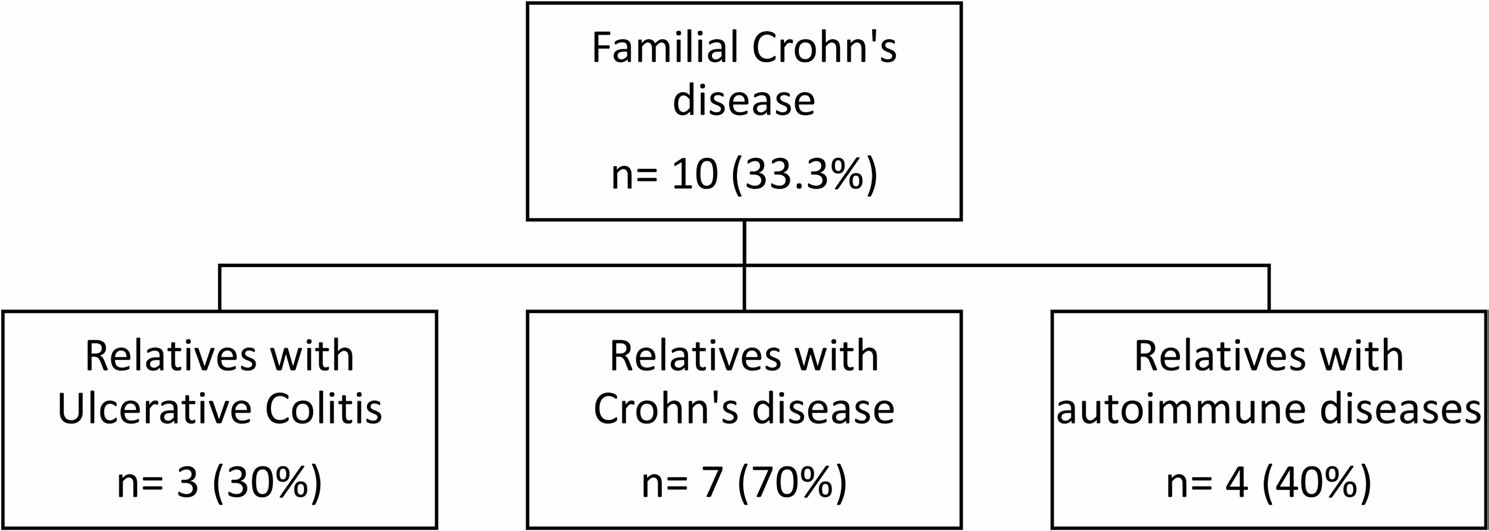
Patients with familial ulcerative colitis

**Fig. 3:**
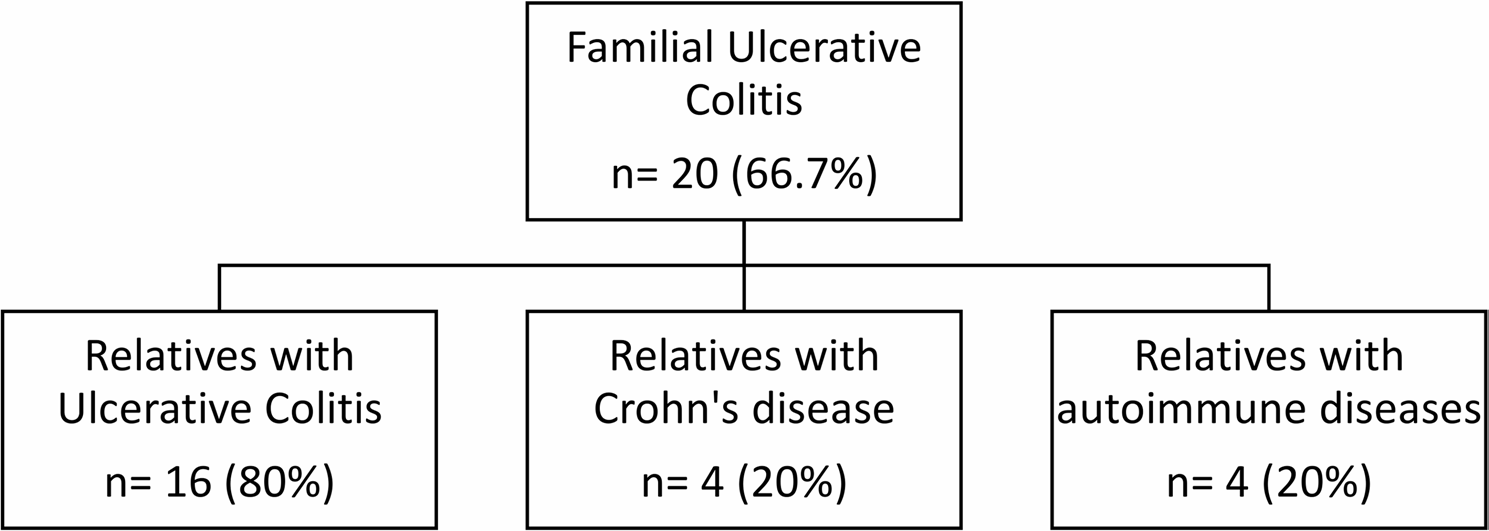
Patients with familial Crohn’s disease

90 patients (30 familial, 60 sporadic) attended the one-year follow-up (Fig. 1). In the familial group, 20 patients were diagnosed with ulcerative colitis (UC) and 10 with Crohn’s disease (CD). Of the 20 familial UC cases, 16 had a first-degree relative diagnosed with UC, 4 had a relative with CD, and 4 had relatives with other autoimmune conditions (Fig. 2; Table 1). Among the 10 familial CD cases, 7 had a first-degree relative diagnosed with CD, 3 had a relative with UC, and 4 had relatives with other autoimmune conditions (Fig. 3; Table 1). The groups matched well in terms of age and sex (*p* > 0.05, Table 1). The marital status significantly differed with more patients who were single in familial cases (60% vs. 28.3%, *p* = 0.012). Furthermore, self-reported intolerance to milk products was significantly more common in familial cases (73.3% vs. 50%, *p* = 0.035, Table 2).

**Table 1:** Demographic data and clinical characteristics of the studied patients

		No. = 90
Age	**Mean ± SD**	33.89 ± 12.13
	**Range**	18–67
Sex	**Male**	41 (45.6%)
	**Female**	49 (54.4%)
Marital status	**Married**	53 (58.9%)
	**Single**	35 (38.9%)
	**Divorced**	2 (2.2%)
Occupation	**Manual**	8 (8.9%)
	**Professional**	22 (24.4%)
	**Not working**	46 (51.1%)
	**Student**	14 (15.6%)
Smoking	**Smoker**	8 (8.9%)
	**Nonsmoker**	76 (84.4%)
	**Ex smoker**	6 (6.7%)
Residence	**Urban**	68 (75.6%)
	**Rural**	22 (24.4%)
Degree of consanguinity	**No consanguinity**	60 (66.7%)
	** 1 st degree**	18 (20.0%)
	**2nd degree**	12 (13.3%)
Cases with related autoimmune disorders	**No**	67 (74.4%)
	**Yes**	23 (25.6%)
	***Rheumatoid***	*8 (34.8%)*
	***SLE***	*8 (34.8%)*
	***Celiac***	*4 (17.4%)*
	***Thyroid***	*3 (13.0%)*
Familial		
Ulcerative [20 (66.7%)]	**Relative Ulcerative**	16 (80.0%)
	**Relative Crohn’s**	4 (20.0%)
	**Relative autoimmune**	4 (20.0%)
Crohn’s [10 (33.3%)]	**Relative Ulcerative**	3 (30.0%)
	**Relative Crohn’s**	7 (70.0%)
	**Relative autoimmune**	4 (40.0%)

**Table 2:** Demographic data and clinical characteristics of Familial cases (group A) and sporadic cases (group B)

		Group A (Familial)	Group B (Sporadic)	Test value	P value
		No.= 30	No.= 60		
Age	**Mean ± SD**	32.1 ± 13.56	34.78 ± 11.36	−0.989•	0.325
	**Range**	18–67	18–61		
Sex	**Male**	15 (50%)	26 (43.3%)	0.358*	0.549
	**Female**	15 (50%)	34 (56.7%)		
Marital status	**Married**	12 (40%)	41 (68.3%)	8.884*	0.012
	**Single**	18 (60%)	17 (28.3%)		
	**Divorced**	0 (0%)	2 (3.3%)		
Occupation	**Manual**	2 (6.7%)	6 (10%)	2.021*	0.568
	**Professional**	10 (33.3%)	12 (20%)		
	**Not working**	14 (46.7%)	32 (53.3%)		
	**Student**	4 (13.3%)	10 (16.7%)		
Smoking	**Smoker**	3 (10%)	5 (8.3%)	0.839*	0.657
	**Nonsmoker**	26 (86.7%)	50 (83.3%)		
	**Ex smoker**	1 (3.3%)	5 (8.3%)		
Residence	**Urban**	21 (70%)	47 (78.3%)	0.752*	0.386
	**Rural**	9 (30%)	13 (21.7%)		
Cases with related autoimmune disorders	**No**	22 (73.3%)	45 (75%)	0.029*0	0.864
	**Yes**	8 (26.7%)	15 (25%)		
	**Rheumatoid**	3 (37.5%)	5 (33.3%)	0.775	0.856
	**SLE**	2 (25.0%)	6 (40.0%)		
	**Celiac**	2 (25.0%)	2 (13.3%)		
	**Thyroid**	1 (12.5%)	2 (13.3%)		
Surgical intervention	**Yes**	6 (20%)	5 (8.3%)	2.537*	0.111
	**No**	24 (80%)	55 (91.7%)		
Psychological disorders	**Yes**	13 (43.3%)	18 (30%)	1.575*	0.21
	**No**	17 (56.7%)	42 (70%)		
Self-reported intolerability to milk products	**Yes**	22 (73.3%)	30 (50%)	4.464*	0.035
	**No**	8 (26.7%)	30 (50%)		
Evident clinical uveitis at any time	**Yes**	5 (16.7%)	19 (31.7%)	2.301*	0.129
	**No**	25 (83.3%)	41 (68.3%)		

•: Independent t-test; *: Chi-square test

Evident clinical uveitis was found in 26.7% of the patients. Psychological disorders were discovered in 34.4% of the patients—approximately 58% of cases self-reported milk product intolerance. Regarding lines of management, 88% of patients were receiving medical treatment, including biological therapy, whereas 12% underwent surgical interventions (Table 2).

At presentation, patients with sporadic UC had more advanced disease compared with those with familial UC. This was reflected in significantly greater Mayo scores (median 11 compared with 8, *p* < 0.001), Mayo endoscopic scores (median 3 compared with 2, *p* = 0.003), as well as a larger proportion as severe according to the Truelove criteria (64.1% compared with 30%, *p* = 0.040, Table 3). Conversely, no significant differences in disease severity at entry occurred for patients with CD. Contrarily, with fewer endoscopic features of disease in UC, the familial patients had significantly greater levels of systemic inflammatory markers at entry, including median ESR, CRP, as well as CRP/albumin ratio (all *p* < 0.05, Table 4).

**Table 3:** Comparison of disease characteristics and use of biological therapy between group A and group B

		Group A	Group B	Test value	P value
		No.= 30	No.= 60		
Ulcerative/Crohn’s	**Ulcerative**	20 (66.7%)	39 (65.0%)	0.025	0.875
	**Crohn’s**	10 (33.3%)	21 (35.0%)		
Disease severity by Truelove criteria	**Mild**	3 (15%)	2 (5.1%)	6.438*	0.040
	**Moderate**	11 (55%)	12 (30.8%)		
	**Severe**	6 (30%)	25 (64.1%)		
Mayo score in ulcerative colitis	**Median (IQR)**	8 (6.5–9)	11 (9–11)	−3.514^≠^	0.000
	**Range**	4–12	4–12		
Mayo endoscopic scoring system	**Median (IQR)**	2 (2–3)	3 (2–3)	−2.922^≠^	0.003
	**Range**	1–3	1–3		
Location of disease in UC	**Rectal**	3 (15%)	2 (5.1%)	2.037*	0.361
	**Left**	7 (35%)	12 (30.8%)		
	**Pancolitis**	10 (50%)	25 (64.1%)		
	**Pouchitis**	0 (0%)	0 (0%)		
	**Ileal affection**	0 (0%)	0 (0%)		
Crohn’s disease activity index	**Median (IQR)**	185 (161–220)	187 (161–351)	−0.782^≠^	0.434
	**Range**	134–329	139–452		
Simplified Crohn’s activity index	**Median (IQR)**	5.5 (5–6)	6 (5–7)	−1.138^≠^	0.255
	**Range**	5–7	5–8		
Location of disease in Crohn’s disease	**Colonic**	1 (10%)	5 (23.8%)	4.814*	0.090
	**Ileal**	1 (10%)	8 (38.1%)		
	**Ileocolonic**	8 (80%)	8 (38.1%)		
Dysplasia	**No**	28 (93.3%)	55 (91.7%)	0.131*	0.937
	**Low grade**	1 (3.3%)	3 (5%)		
	**High-grade adenocarcinoma**	1 (3.3%)	2 (3.3%)		
Use of biological therapy	**Yes**	18 (60%)	24 (40%)	3.214*	0.073
	**No**	12 (40%)	36 (60%)		
Type of used biological therapy	**Infliximab**	8 (42.1%)	9 (37.5%)	0.774*	0.979
	**Adalimumab**	6 (31.6%)	8 (33.3%)		
	**Ustekinumab**	1 (5.3%)	3 (12.5%)		
	**Infliximab then Ustekinumab**	2 (10.5%)	2 (8.3%)		
	**Infliximab then adalimumab**	1 (5.3%)	1 (4.2%)		
	**Adalimumab then Ustekinumab**	1 (5.3%)	1 (4.2%)		
Time from starting first biologics (years)	**Mean ± SD**	4.47 ± 3.19	3.27 ± 1.88	1.543	0.131
	**Range**	1–13	0.5–7		
Cause of using biological therapy	**Steroid dependent**	8 (42.1%)	11 (47.8%)	3.928*	0.269
	**Steroid resistant**	6 (31.6%)	9 (39.1%)		
	**Extraintestinal**	2 (10.5%)	3 (13%)		
	**Fistula**	3 (15.8%)	0 (0%)		

*: Chi-square test; ≠: Mann-Whitney test

**Table 4:** Comparison between group A and group B regarding inflammatory markers at diagnosis and after one year

Lab at diagnosis		Group A	Group B	Test value	*P* value
		No.= 30	No.= 60		
ESR (0–15)	**Median (IQR)**	23 (20–41)	10 (4–26.5)	−3.398≠	0.001
	**Range**	2–73	1–98		
CRP (0.3–1.3)	**Median (IQR)**	48 (32–68)	30 (7–55)	−2.077≠	0.038
	**Range**	1.9–128	2–100		
CRP/Albumin	**Median (IQR)**	13.88 (8.57–19.5)	7.92 (1.71–16.77)	−2.294≠	0.022
	**Range**	0.38–34.13	0.53–28.71		
Fecal calprotectin at diagnosis (less than 10)	**Median (IQR)**	652.5 (325–846)	499.5 (290.5–656.5)	−1.537	0.124
	**Range**	139–1324	30–1333		
Lab at one year					
ESR (0–15)	**Median (IQR)**	12 (5–25)	4 (2.5–17.5)	−3.182≠	0.001
	**Range**	2–60	2–40		
CRP (0.3–1.3)	**Median (IQR)**	5 (3–12)	13.5 (5–43.5)	−1.977≠	0.048
	**Range**	0.4–160	2–88		
CRP/Albumin	**Median (IQR)**	1.41 (0.81–3.43)	3.68 (1.27–11.83)	−1.939≠	0.053
	**Range**	0.1–44.44	0.5–22.89		
Fecal calprotectin after one year (less than 10)	**Median (IQR)**	138 (79–402)	202 (166–360.5)	−2.530≠	0.011
	**Range**	25–1346	120–763		

IQR: Interquartile range; ≠: Mann-Whitney test

**Fig. 4:**
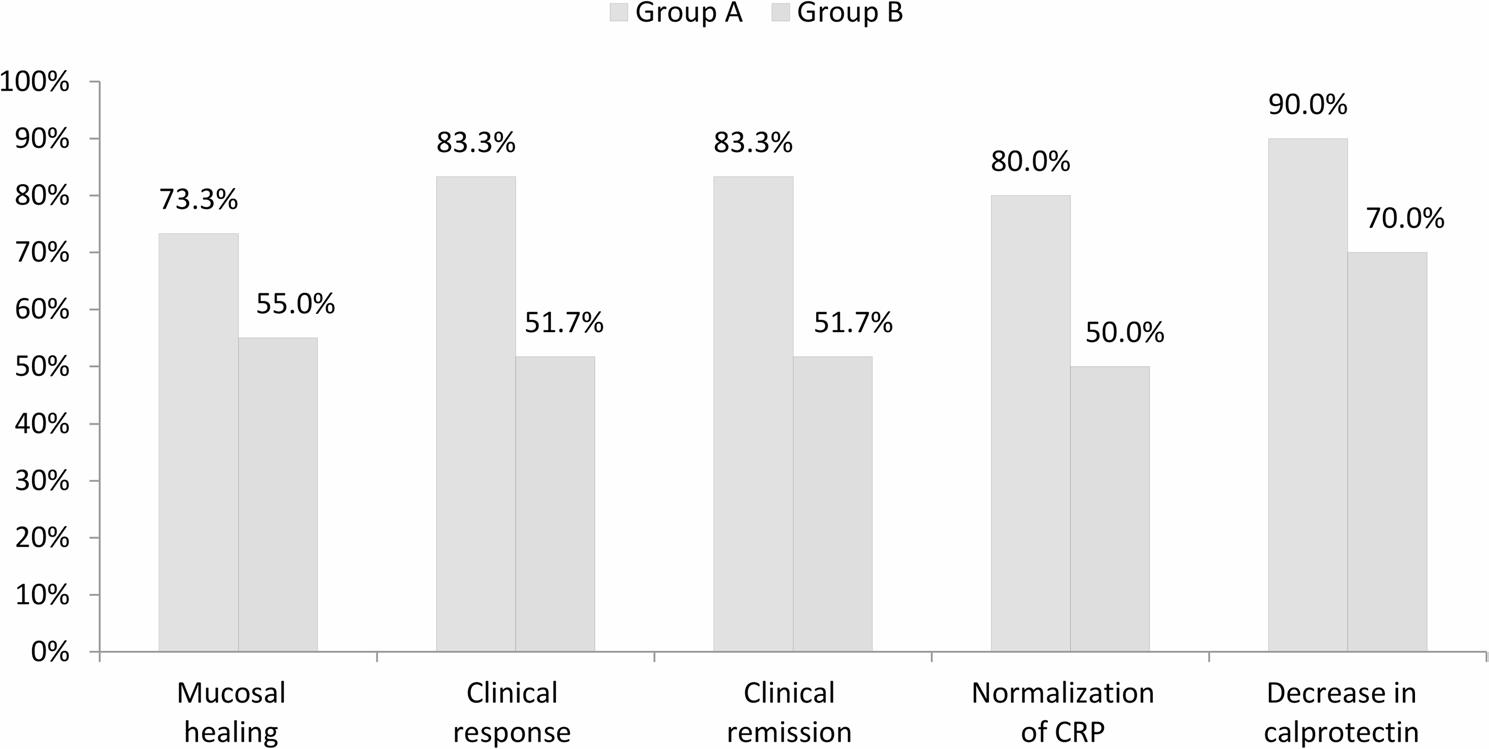
Comparison of outcomes between group A and group B after 1 year

**Fig. 5:**
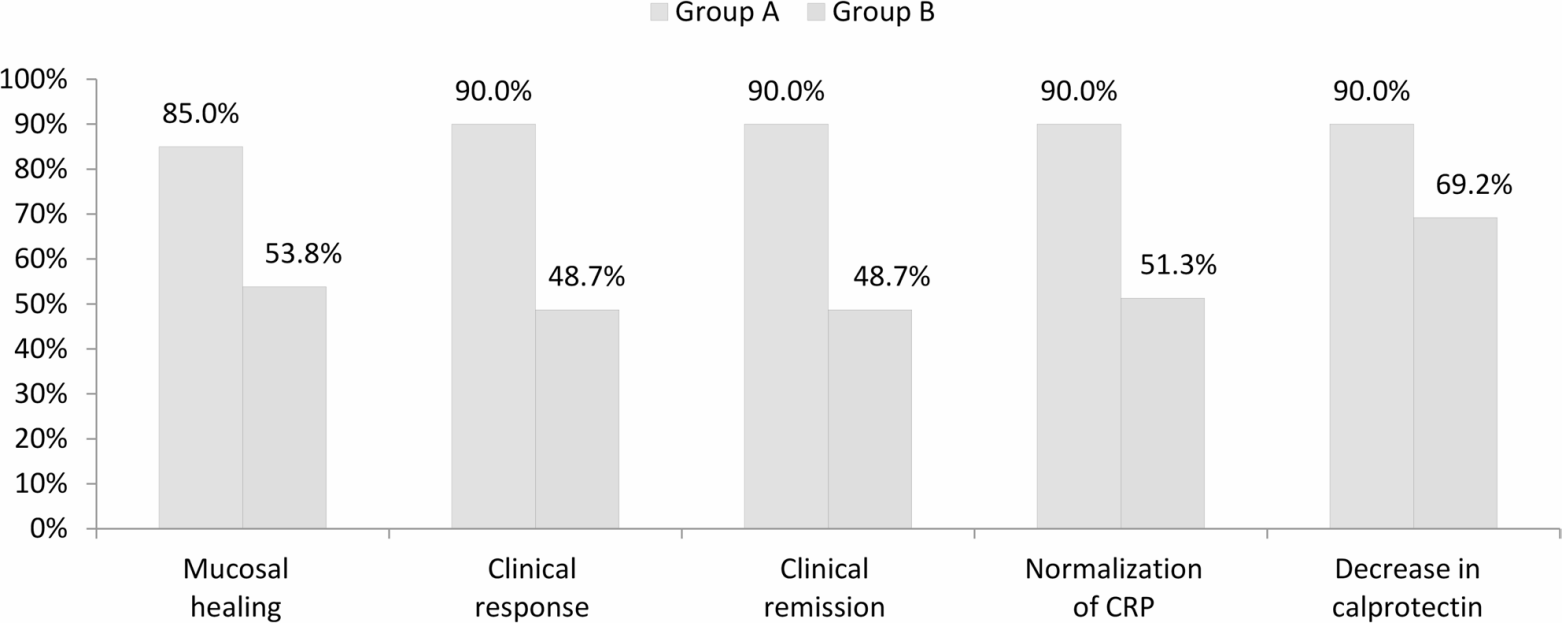
Comparison of outcomes between familial ulcerative and sporadic ulcerative patients

Following one year of therapeutic intervention, patients with familial inflammatory bowel disease (IBD) exhibited consistently improved clinical outcomes when compared to those with sporadic cases. These familial patients realized markedly higher rates of clinical response (83.3% versus 51.7%, *p* = 0.003), clinical remission (83.3% versus 51.7%, *p* = 0.003), and normalization of C-reactive protein (CRP) levels (80% versus 50%, *p* = 0.006, Table 5; Fig. 4). A pronounced trend indicating increased mucosal healing was identified within the familial cohort (73.3% versus 55%, *p* = 0.093). The observed treatment advantages were primarily attributed to the ulcerative colitis (UC) subgroup, wherein familial patients demonstrated significantly elevated rates of mucosal healing, clinical response, remission, and CRP normalization (all *p* < 0.05, Table 6; Fig. 5). Additionally, the median percentage reductions in CRP levels, the CRP/albumin ratio, and fecal calprotectin concentrations were significantly greater within the familial group (all *p* < 0.05), signifying a more vigorous inflammatory response to the administered therapy.

**Table 5:** Comparison between group A and group B regarding the outcomes of the studied patients after 1 year

		Group A	Group B	P value	OR (95% CI)
		No.= 30	No.= 60		
Mucosal healing at 1 year	Yes	22 (73.3%)	33 (55%)	0.093	*Ref.*
	No	8 (26.7%)	27 (45%)		2.250 (0.865 to 5.852)
Clinical response at 1 year	Yes	25 (83.3%)	31 (51.7%)	0.003	*Ref.*
	No	5 (16.7%)	29 (48.3%)		4.677 (1.5797 to 13.8499)
Clinical remission at 1 year	Yes	25 (83.3%)	31 (51.7%)	0.003	*Ref.*
	No	5 (16.7%)	29 (48.3%)		4.677 (1.5797 to 13.8499)
Normalization of CRP at 1 year	Yes	24 (80%)	30 (50%)	0.006	*Ref.*
	No	6 (20%)	30 (50%)		4.000 (1.4311 to 11.180)
Decrease in calprotectin at 1 year	Yes	27 (90.0%)	42 (70.0%)	0.034	*Ref.*
	No	3 (10.0%)	18 (30.0%)		3.857 (1.0361 to 14.358)

OR: odds ratio; CI: confidence interval

**Table 6:** Comparison of outcomes between Familial ulcerative and sporadic ulcerative patients

		Group A (Ulcerative)	Group B (Ulcerative)	Test value	P value
		No. = 20	No. = 39		
Mucosal healing at 1 year	Yes	17 (85%)	21 (53.8%)	5.597*	0.018
	No	3 (15%)	18 (46.2%)		
Clinical response at 1 year	Yes	18 (90%)	19 (48.7%)	9.635*	0.002
	No	2 (10%)	20 (51.3%)		
Clinical remission at 1 year	Yes	18 (90%)	19 (48.7%)	9.635*	0.002
	No	2 (10%)	20 (51.3%)		
Normalisation of CRP at 1 year	Yes	18 (90%)	20 (51.3%)	8.645*	0.003
	No	2 (10%)	19 (48.7%)		
Decrease in calprotectin at 1 year	Yes	18 (90.0%)	27 (69.2%)	3.151*	0.076
	No	2 (10.0%)	12 (30.8%)		

*P* value > 0.05: not significant; P value < 0.05: significant; *P* value < 0.01: highly significant

*: Chi-square test

There was a statistically significant increase in the median percentage of reduction in CRP in group A [−86.19 (−92.86 - −24.24)] compared with that in group B [−22.5 (−87.78–104.76)], with a p value = 0.005. Additionally, the median percentage of reduction in the CRP/albumin ratio was significantly greater in group A [−88.77 (−92.24 - −24.24)] than in group B [−15.54 (−87.94−107.05)], with a p value = 0.004, and the median percentage of reduction in the faecal calprotectin level was significantly greater in group A [−77.31 (−88.3–1.66)] than in group B [−45.29 (−74.83–21.07)], with a p value = 0.010 Table 7.

**Table 7:** Comparison of the percentages of change in the studied parameters between group A and group B

		Group A	Group B	Test value	P value
		No. = 30	No. = 60		
Albumin (3.4–5.4)	Median (IQR)	7.11 (0–20)	0 (−6.52–13.8)	−1.774≠	0.076
	Range	−20.83–66.67	−22.22–50		
	Range	−17.07–28.57	−29.57–27.27		
ESR (0–15)	Median (IQR)	−56.19 (−75–5)	−35.42 (−83.33–26.67)	−0.484≠	0.629
	Range	−96.08–800	−96.49–566.67		
CRP (0.3–1.3)	Median (IQR)	−86.19 (−92.86 - −24.24)	−22.5 (−87.78–104.76)	−2.821≠	0.005
	Range	−98.72–792.86	−97.06–1600		
CRP Albumin	Median (IQR)	−88.77 (−92.24 - −24.24)	−15.54 (−87.94−107.05)	−2.910≠	0.004
	Range	−98.49–722.37	−96.89–1330.73		
Fecal calprotectin	Median (IQR)	−77.31 (−88.3–1.66)	−45.29 (−74.83–21.07)	−2.568≠	0.010
	Range	−96.93–320.41	−86.8–746.67		

≠: Mann-Whitney test

Univariate logistic regression analysis of factors associated with mucosal healing in the familial group revealed that platelet counts ≤ 324 and faecal calprotectin levels ≤ 679 were significantly associated with mucosal healing among patients in group A, with p-values of 0.012 and 0.008, respectively. Additionally, the multivariate logistic regression analysis revealed that the most prominent factor associated with mucosal healing among patients in group A was a platelet level ≤ 324, with an OR (odds ratio) (95% CI (confidence interval)) of 18.667 (1.879–185.399) and a p value = 0.012 (Table 8).

**Table 8:** Univariate and multivariate logistic regression analyses of factors associated with mucosal healing among patients in group A

	Univariate				Multivariate			
	*P* value	OR	95% C.I. for OR		*P* value	OR	95% C.I. for OR	
Lower			Upper	Lower			Upper	
Psychological factors	0.631	1.247	0.507	3.068	-	-	-	-
Platelet ≤ 324	0.012	18.667	1.879	185.399	0.012	18.667	1.879	185.399
Fecal calprotectin ≤ 679	0.008	13.500	1.955	93.246	-	-	-	-
Crohn’s	0.052	5.667	0.990	32.428	-	-	-	-

*OR* odds ratio, *CI* confidence interval

In the sporadic group, univariate logistic regression analysis revealed that faecal calprotectin levels ≤ 406 and receiving biologic treatment were significantly associated with mucosal healing among patients in group B, with p-values of < 0.001 and 0.013, respectively. Additionally, the multivariate logistic regression analysis revealed that the most important factor associated with mucosal healing among patients in group B was a faecal calprotectin level ≤ 406, with an OR (95% CI) of 7.980 (2.148–29.644) and a p value = 0.002 (Table 9).

**Table 9:** Univariate and multivariate logistic regression analyses of factors associated with response among patients in group B

	Univariate				Multivariate			
	*P* value	OR	95% C.I. for OR		*P* value	OR	95% C.I. for OR	
Lower			Upper	Lower			Upper	
Fecal calprotectin ≤ 406	0.000	10.062	2.806	36.079	0.002	7.980	2.148	29.644
Biologics	0.013	4.200	1.347	13.093	0.135	2.633	0.739	9.381

*OR* odds ratio, *CI* confidence interval

### When the familial group was stratified by the degree of relatedness, we compared the three groups and found that

Sporadic ulcerative colitis patients of Group B showed significantly increased disease severity on presentation compared with both first- and second-degree familial subgroups according to Mayo and endoscopic scoreing systems (*p* < 0.05 for all). Baseline severity was not significantly different within familial subgroups or in the Crohn’s population cohort. The time to commencing initial biologic therapy was significantly delayed in the first-degree relative subgroup compared with both second-degree and sporadic subgroups (Supplementary Table S1).

**Fig. 6:**
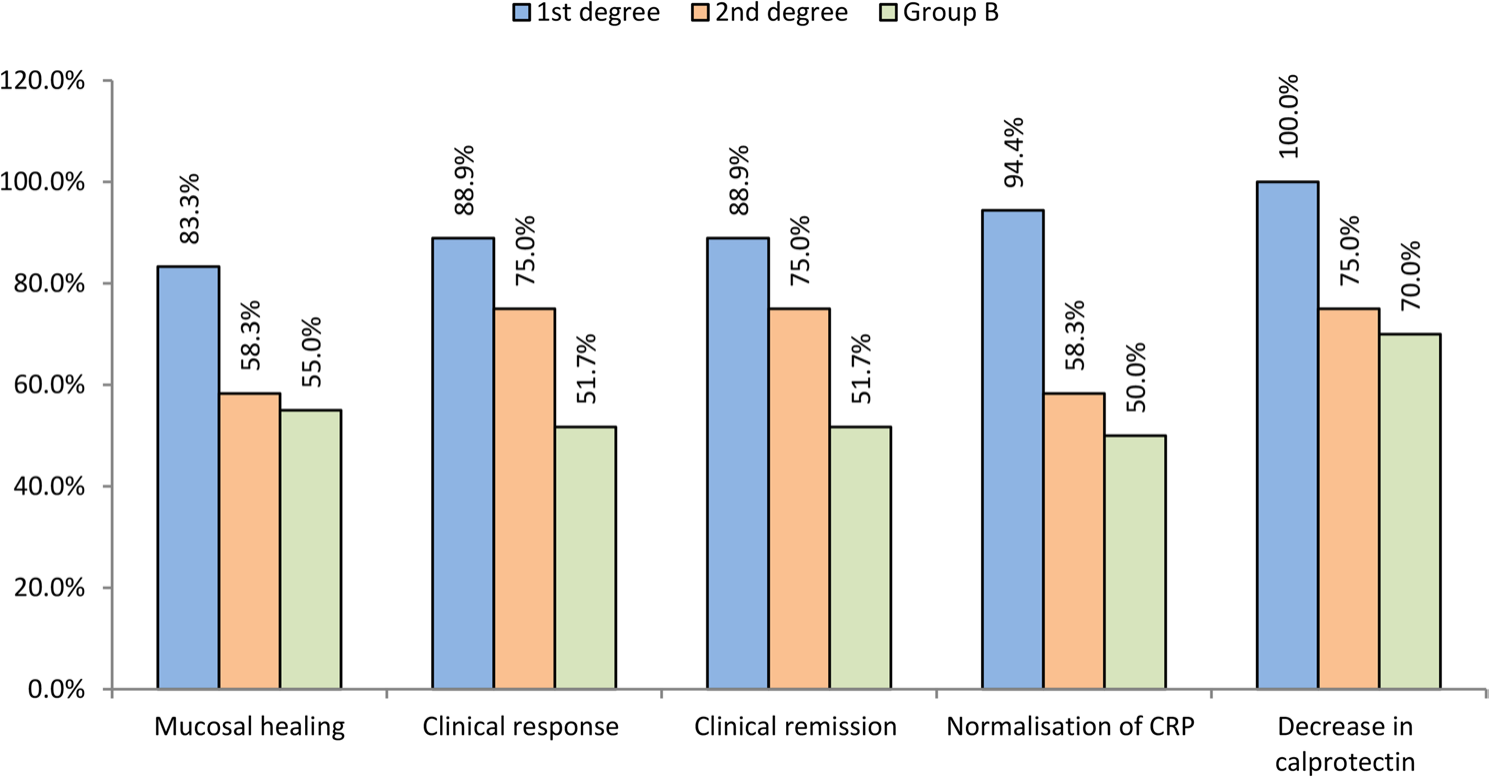
Comparison of outcomes between the three groups after 1 year

The recent comparison reveals that patients with first-degree relatives primarily drive the outcomes identified in the broader familial group. The first-degree group achieved significantly higher levels of clinical response (88.9% vs. 51.7%; *P* = 0.005) and clinical remission (88.9% vs. 51.7%; *P* = 0.005). This response also became evident in the treatment of inflammatory markers. The first-degree group had a significantly higher incidence of CRP normalisation compared to both the second-degree group (94.4% vs. 58.3%, *P* = 0.015) and Group B (94.4% vs. 50.0%, *P* = 0.001). Also, all patients in the first-degree group (100.0%) showed a decrease in calprotectin, with rates significantly higher compared to both the second-degree group (75.0%, *P* = 0.025) and group B (70.0%, *P* = 0.008). While the second-degree relative group showed a clear tendency for better outcomes compared with group B, it didn’t reach a statistically significant difference (Supplementary Table S2, Fig. 6).

**Fig. 7:**
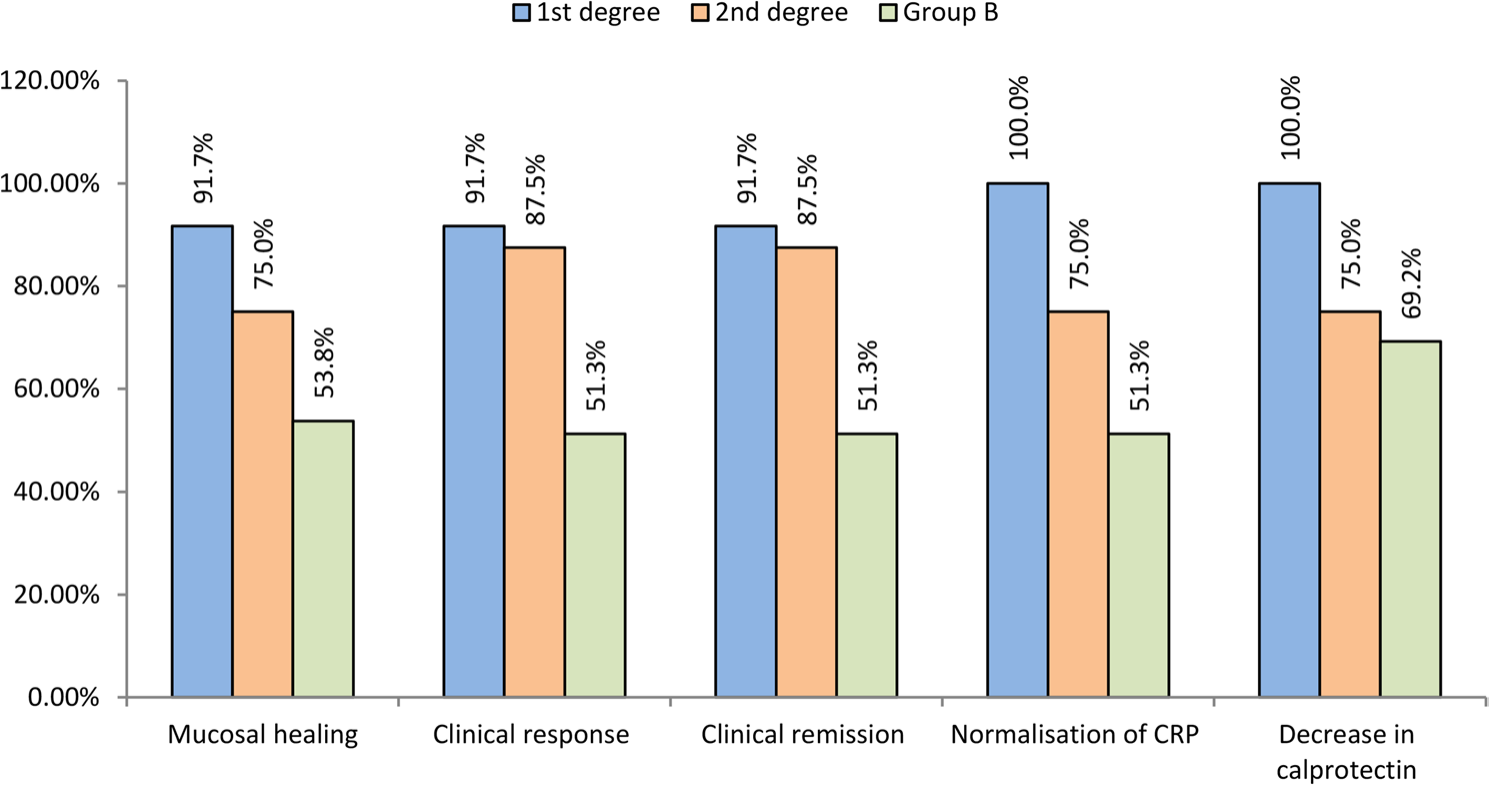
Comparison of outcomes between familial ulcerative and sporadic ulcerative patients

Furthermore, subgroup analysis of ulcerative colitis revealed that patients with a first-degree relative demonstrated significantly better outcomes than patients with sporadic cases of UC. The subgroup with a first-degree relative demonstrated significantly increased mucosal healing (91.7% versus 53.8%; *P* = 0.017), clinical response (91.7% versus 51.3%; *P* = 0.012), and clinical remission (91.7% versus 51.3%; *P* = 0.012). In addition, a highly significant difference was revealed in CRP normalization with 100% of the subgroup with a first-degree relative achieving normalization compared with 51.3% of the subgroup with sporadic cases (*P* = 0.002). The subgroup with second-degree relatives demonstrated improved outcomes that did not reach statistical significance compared with both the subgroup with a first-degree relative as well as with those with sporadic cases (Fig. 7).

## Discussion

Inflammatory bowel disease (IBD) has emerged as a significant health concern in the Middle East over the past decade, with a noticeable increase in its incidence and progression. Despite this growing trend, a lack of comprehensive studies remains in the region, particularly in Egypt, regarding the nature of the disease and its genetic predisposition. Clinically, we have observed an increasing pattern of familial aggregation, particularly among first-degree relatives; however, no strong evidence-based studies have yet confirmed this trend in our region. Motivated by these observations, we initiated an observational study within our IBD patient group at Ain Shams University Hospitals, Cairo, Egypt, to assess the prevalence of familial clustering. To our astonishment, a substantial percentage of patients had first- or second-degree relatives affected by the disease, reinforcing the need for a deeper understanding of its hereditary factors.

This study aimed to evaluate the clinical characteristics of IBD patients with a family history of the disease and compare them to sporadic cases in terms of disease phenotype, progression, natural history, clinical presentation, severity, and treatment response. Understanding these differences could provide crucial insights into the genetic and environmental factors that influence IBD in our population.

Traditionally, most IBD occurs in people aged 15–30 years, and up to 25% of patients will develop IBD by adolescence. There appears to be a bimodal distribution, with a second peak of 10% to 15% developing IBD after age 60 [19].

The demographic profile of our cohort fits with known patterns in Egyptian IBD patients. The mean age at presentation (33.89 ± 12.13 years) is in agreement with earlier Egyptian research with ages of 28.5 to 35.1 years [2, 20, 21]. As with overseas notes from Chung et al. [15], presenting age was younger in patients with Crohn’s disease (familial CD: 24.1 ± 7.37 years) than with ulcerative colitis patients (familial UC: 36.1 ± 14.29 years). Early presentation in CD might represent both genuine biologic patterns as well as enhanced detection capacity due to improved methods [19].

In terms of sex distribution, our study exhibited a slight predominance of females (54.4%), with a male-female ratio of 1:1.195, which is in agreement with other Egyptian cohorts with reported ratios of 1:1.16 to 1:1 [20, 21]. This even distribution among both familial cases and sporadic cases implies no substantial sex-linked predisposition within our population. Other studies reported a similar prevalence in both sexes [22, 23]. Additionally, Elbadry et al. [2] reported that the sex distribution was almost equal, with a significant male dominance (60.4%, *p* = 0.003) among patients with CD. The sex-specific distributions of CD and ulcerative colitis depend, among other things, on ethnicity. Although women in Europe and the USA are affected twice as often as men are affected by Crohn’s disease, in Asia, more men are affected than women. In UC, there is no difference in incidence related to sex or continent [23].

Our study revealed that most patients in both familial and sporadic cases were living in urban areas. Increased urbanisation is one hypothesis for the increasing incidence of IBD. The most extensive epidemiological study on IBD in Egypt revealed that more than half of the study population (57%) resided in rural areas [2], which is inconsistent with the available literature on IBD demographics. The urban population in Egypt has remained relatively stable since 2010, accounting for 42.8% of the Egyptian population. However, lifestyles in rural areas have urbanised, and this change should be studied selectively [2]. Urbanisation impacts the gut microbiota through the westernisation of diets that are low in carbohydrates and high in animal proteins and fats, increased pollution levels, increased antibiotic usage, and improved hygiene status. These factors alter the gut microbiota, which is considered a contributing factor to the development of IBD [24].

Smoking is an important environmental factor in inflammatory bowel disease (IBD), with different effects on ulcerative colitis (UC) and CD. Never smoking and former smoking increase the risk of UC, whereas smoking exacerbates the course of CD [25].

In our study, the majority of patients in both groups were nonsmokers. Similar percentages were reported in other Egyptian studies [2, 20]. In our unique study, no significant difference was noted between familial and sporadic IBD patients in terms of smoking status.

Our study revealed that milk consumption may be related to disease severity, as the percentage of self-reported milk intolerability was higher in familial cases than in sporadic cases.

This finding aligns with a study conducted by Komperød et al., which investigated the role of diet in maintaining remission in patients with Crohn’s disease, involving 16 patients. Dairy was reported as the most frequent dietary trigger of symptoms. All symptoms improved (*p* < 0.05) during the elimination diet period, especially in patients with small intestinal effects. Cow’s milk is the most commonly reported dietary trigger of gastrointestinal symptoms, such as abdominal pain, flatulence, bloating, foul-smelling wind/faeces, and diarrhoea [26]. Vagianos et al. [27] noted that milk and milk products exacerbate gastrointestinal symptoms and are thus avoided by IBD patients. The association of IBD with other diseases, such as lactose intolerance (LI), may play a role. IBD patients have a 2.7-fold greater risk of lactose intolerance. Screening for LIs in this population is warranted to avoid confusing or overlapping symptoms [28]. The potential involvement of milk is debated based on experimental studies that have demonstrated it may cause microbiome alterations and influence intestinal permeability [29].

Studies that assess the associations between milk and dairy consumption and the onset and course of IBD are characterised by significant heterogeneity. Moreover, many studies are of low quality. Taking these aspects into account, it is impossible to draw firm conclusions. In our study, the consumption of milk and dairy products had a greater influence on disease severity, especially in the familial group.

Several scores are used to assess the severity of IBD. The Truelove-Witts and Mayo score classification of disease severity has been the basis of severity index scoring in ulcerative colitis [30]. The CDAI has been commonly used to assess the effects of treatment with different agents in patients with CD [31].

There is insufficient evidence regarding the differences between familial and sporadic cases in terms of disease severity at onset and response to various treatment modalities.

We obtained unique findings in our study; at diagnosis, there was a statistically significant increase in the median ESR, CRP level and CRP/albumin ratio in familial cases. High levels of inflammatory markers at diagnosis in the familial group may reflect the severity of disease onset in such a group of patients. However, the median Mayo score in ulcerative colitis patients was significantly higher in sporadic patients than in familial patients at diagnosis, with a p-value of < 0.001. Additionally, the Mayo endoscopic scoring system was substantially higher in sporadic patients than in familial patients at diagnosis, with a p-value of 0.003. Additionally, the percentage of severely ill patients, according to the TrueLove criteria, was significantly greater in sporadic cases than in familial cases at diagnosis, with a p-value of 0.039.

Despite several studies about the clinical phenotypes of familial IBD, there are still inconsistent and inconclusive results. In many reports, the location, disease severity, disease behaviour, and extraintestinal manifestations of CD are not significantly different between familial and sporadic disease [7, 10, 15, 32]. Additionally, a study conducted by Chung et al. [15] revealed that at diagnosis, the median Mayo score in familial cases was 3.6, whereas it was 3.7 in sporadic cases. For Crohn’s disease, the median CDAI was 120.5 for familial cases and 107.8 for nonfamilial cases. Similarly, Cabré et al. were unable to demonstrate any relevant concordance for the severity items assessed in both CD and UC [33]. However, contrary to these reports, some differences have been noted between familial and sporadic CD. A study conducted by Hwang et al. on 6071 cases of IBD in Korea revealed a more aggressive clinical course of CD in familial cases than in sporadic cases [34].

The Egyptian UC patients with familial inheritance had a superior long-term prognosis compared with some Asian studies, such as those by Dong et al. [33], who recognised an aggressive phenotype for familial CD in East China, and Chung et al. [19], who reported no significant difference between familial and sporadic inflammatory bowel disease (IBD) in South Korea. Differences between our study and those findings could reflect variations in the genetic structures of the studied populations, differences in environmental exposures—such as dietary factors and infections—means of ascertainment, and differences in access to healthcare facilities and treatment modalities. Our findings align with the variability reported in the Arabian area by Mosli et al. [34], supporting the need for larger, regionally representative studies to clarify these associations. After one year, there was a statistically significant decrease in the median levels of CRP and faecal calprotectin in familial cases compared with those in sporadic cases (p values = 0.048 and 0.011, respectively), which may reflect a better response to treatment options in such patients than in sporadic cases.

There is a notable disparity between symptomatology and disease activity in a considerable proportion of patients with inflammatory bowel disease (IBD), and escalation of treatment based on symptoms alone can fail to alter the course of disease significantly. The STRIDE-II position statement, published in 2021 by the Selecting Therapeutic Targets in Inflammatory Bowel Disease (STRIDE) initiative of the IOIBD, provides the most current guidelines for a treat-to-target (T2T) approach in IBD [35].

We found that patients with familial ulcerative colitis had a greater clinical response, remission, and normalisation of CRP after one year, with p-values of 0.018, 0.002, 0.002, and 0.003, respectively, compared to patients with sporadic ulcerative colitis. Moreover, the percentage of patients with a decrease in faecal calprotectin at one year was significantly greater in familial cases than in sporadic cases, with a p-value = 0.034.

The improved therapy outcomes in familial IBD patients, while not as a result of differential usage of biological therapy (Table 3), may be due to various favourable factors. Familial cases may owe benefit through greater awareness of illness, with attendant earlier presentation and therapy. Furthermore, common familial experience with IBD may promote greater compliance with therapy along with greater anticipatory healthcare seeking. The clinical advantage accruing from these attributes, as well as potential biological differences as a result of common genes as well as common environment, may collectively explain improved outcome in familial IBD patients.

In Egypt, the prevailing first-started biological treatments for IBD, under government and insurance programs are anti-tumour necrosis factor (anti-TNF) medications such as Infliximab and Adalimumab. For instance, it has been found that more than half the number of IBD sufferers treated biologically in a study carried out in Egypt were treated by anti-TNF [36].

In many IBD units, the first line biological agent used can depend largely on certain factors that include disease activity and type (e.g., fistulizing, stricturing Crohn’s disease versus severe Ulcerative Colitis), and the presence or absence of Extra-intestinal manifestations, tolerability, and availability. Thus, individuals with severe Crohn’s disease, for example, may start first-line therapy using anti TNF monoclonal antibodies, given proven efficacy for such scenarios. Decisions about biological therapy initiation include assessment of levels of biomarkers (CRP, Calprotectin), endoscopic activity, existing arthropathies and skin manifestations (like psoriasis). Rather, the algorithms used for concomitant therapies will depend largely on “first line TNF antagonists, then class switching if failure,” especially when resources are limited [37].

As part of patient follow-up at our centre, a colonoscopy is performed one year after the start of treatment for assessment purposes. Our study revealed that 55 patients (61.1%) experienced mucosal healing after one year of therapy, comprising 38 patients with ulcerative colitis and 17 patients with Crohn’s disease. Twenty-two (73.3%) familial patients experienced mucosal healing, whereas thirty-three (55%) sporadic patients experienced mucosal healing after one year. One of the unique findings in our study was that the percentage of patients with mucosal healing at one year was significantly higher in familial ulcerative patients than in sporadic ulcerative patients, with a p-value of 0.018.

Additionally, the percentage of mucosal healing in familial Crohn’s disease patients was greater than that in sporadic Crohn’s disease patients; however, this difference was not statistically significant. This may be because the prevalence of Crohn’s disease in our study was lower than that in patients with ulcerative colitis.

Identifying predictors of mucosal healing (MH) can guide treatment strategies and improve patient outcomes. The key predictors included clinical characteristics, disease extent and duration, as well as the levels of CRP and faecal calprotectin. Patients with less extensive disease and a shorter disease duration tend to achieve MH more readily. Conversely, extensive disease and longer disease duration may be associated with a lower likelihood of achieving MH [38]. The presence of fever at diagnosis in patients with Crohn’s disease and medical treatment without steroids have been identified as significant predictors for achieving MH [39].

In this study, we found that platelet counts ≤ 324 and faecal calprotectin levels ≤ 679 in familial cases were significantly associated with mucosal healing among patients with familial cases, with p-values of 0.012 and 0.008, respectively. The most important factor associated with mucosal healing among familial cases was a platelet level ≤ 324. In sporadic cases, faecal calprotectin ≤ 406 and receiving biologic treatment were significantly associated with mucosal healing, with p-values < 0.001 and 0.013, respectively. Additionally, the most crucial factor associated with mucosal healing among sporadic cases was the faecal calprotectin level. This finding was in accordance with that of Nakarai et al., who studied 43 patients and reported that a low PLT decreases the risk of relapse in ulcerative colitis patients with mucosal healing [40]. Similarly, a study conducted by Furukawa et al. on 345 Japanese patients revealed that the PLT was independently inversely associated with mucosal healing [41]. Additionally, a study conducted by Cao et al. revealed that faecal calprotectin is a reliable non-invasive biomarker for predicting complete mucosal healing in patients with ulcerative colitis [42]. Additionally, Alain et al., who studied 228 ulcerative colitis patients, reported that the level of faecal calprotectin was better correlated with endoscopic disease activity than with clinical activity, CRP, platelets, haemoglobin, and blood leukocytes. The strong correlation with endoscopic disease activity suggests that faecal calprotectin represents a valuable biomarker for non-invasive monitoring of disease activity in UC patients [43].

A study by Trier Moller et al. [44] also reported an increased rate of surgery among familial CD patients after 2 years of disease duration and a slightly shorter time to first anti-tumour necrosis factor-alpha (TNF-α) therapy among familial CD and UC patients compared to sporadic patients. Nonetheless, these findings may reflect variations in care, which could be driven by patients or doctors being more proactive in treatment as a result of their perception of the disease course in their relatives. In our study, 12.22% of patients needed surgery, whereas 87.78% did not. The indications for surgery differ. Three patients required it because of acute severe ulcerative colitis; five patients did not respond to medical treatment, and three had fistulae. Regarding the need for surgery in familial cases, six patients required surgery, whereas in five sporadic cases, we found no statistically significant difference, with a p-value of 0.111.

The primary limitation of the study is its small sample size, particularly in the patient groups for Crohn’s disease and second-degree relatives, which limits the power of the statistics to demonstrate differences in the present study. A post-hoc power analysis confirmed that the study was adequately powered (> 80%) for the primary comparison between familial and sporadic IBD. However, the subgroup analysis for Crohn’s disease was underpowered due to the limited number of familial CD cases (*n* = 10). Therefore, the CD findings should be considered exploratory and interpreted with caution, as they may represent true null findings or reflect limited power to detect modest effects.

The paper contributes useful regional information. To the best of our knowledge, this is the first comparison between sporadic and familial IBD in terms of their clinical features and therapeutic outcomes, directly in the Egyptian context. While earlier Egyptian studies, as well as those in the larger Middle Eastern region, had focused on overall epidemiology without differentiating between non-familial and familial IBD, the present work, therefore, helps fill a crucial regional knowledge gap for the first time, where the number of IBD cases continues to increase. Although our study sample size was small, it had new findings with statistical significance regarding disease course and treatment response.

Understanding these differences could provide crucial insights into the genetic and environmental influences on IBD within our population, highlighting the necessity for region-specific studies.

Given the observed familial clustering, several essential questions arise: Should we implement routine screening protocols for asymptomatic family members of individuals with IBD? Would biomarkers such as C-reactive protein (CRP) or faecal calprotectin be valuable tools for early detection? Moreover, should non-invasive imaging modalities such as bowel ultrasound or enterography or even invasive colonoscopy, be considered for family members who present with irritable bowel syndrome (IBS)-like symptoms, such as diarrhoea or abdominal pain?

Furthermore, should dedicated screening clinics for at-risk family members be established, like the specialised clinics currently available for colorectal cancer, hepatitis B, or hepatitis C? Addressing these questions is crucial for early diagnosis and potential preventive strategies. As familial patterns of IBD become increasingly evident, a structured approach to screening and early intervention may significantly impact disease outcomes in our population.

## Conclusions

Despite the limited number of studies in the Middle East, familial IBD patients may present with a more severe initial inflammatory response but demonstrate better long-term treatment outcomes. These findings suggest that genetic and environmental influences play a role in IBD, highlighting the need for region-specific studies.

## Supplementary Information

Supplementary Material 1. Table S1. Subgroup analyses: demographic, clinical and treatment characteristics comparing first-degree relatives, second-degree relatives and sporadic cases

Supplementary Material 2. Table S2. Subgroup outcomes: clinical response, remission and time-to-biologic in first-degree, second-degree and sporadic cases

## Data Availability

The datasets used are available from the corresponding author upon reasonable request.

## Abbreviations

ALT: Alanine aminotransferase
AST: Aspartate aminotransferase
CBC: Complete blood count
CD: Crohn’s Disease
CDAI: Crohn’s Disease Activity Index
CI: Confidence interval
CRP: C-reactive protein
ESR: Erythrocyte sedimentation rate
GI: Gastrointestinal
IBD: Inflammatory Bowel Disease
IOIBD: International Organisation for the Study of Inflammatory Bowel Disease
IQR: Interquartile range
LI: Lactose intolerance
MH: Mucosal Healing
NSAID: Nonsteroidal Anti-Inflammatory Drug
OR: Odds ratio
SD: Standard deviation
STRIDE: Selecting Therapeutic Targets in the Inflammatory Bowel Disease Initiative
T2T: Treat-to-Target
TNF-α: Tumour necrosis factor-alpha
UC: Ulcerative Colitis
