# Supplementary material for: Clinical characteristics of familial and sporadic inflammatory bowel disease in Egyptian patients
Source: BMC Gastroenterol. 2025 Dec 15;26:43. doi: 10.1186/s12876-025-04492-9 (PMC12821981; doi:10.1186/s12876-025-04492-9)
Supplement: Supplementary file 1 — Supplementary Material 1. Table S1. Subgroup analyses: demographic, clinical and treatment characteristics comparing first-degree relatives, second-degree relatives and sporadic cases [file 12876_2025_4492_MOESM1_ESM.docx]

**Table 10:** Comparison of disease characteristics and use of biological therapy between 1^st^ degree relatives, 2^nd^ degree relatives and group B

|  | | **1st degree** | **2nd degree** | **Group B** | **Test value** | **P-value** | **P1** | **P2** | **P3** |
| --- | --- | --- | --- | --- | --- | --- | --- | --- | --- |
|  |  | **No. = 18** | **No. = 12** | **No. = 60** |  |  |  |  |  |
| Ulcerative/Crohn's | Ulcerative | 12 (66.7%) | 8 (66.7%) | 39 (65.0%) | 0.025* | 0.988 | - | - | - |
|  | Crohn's | 6 (33.3%) | 4 (33.3%) | 21 (35.0%) |  |  |  |  |  |
| Disease severity by Truelove criteria | Mild | 2 (16.7%) | 1 (12.5%) | 2 (5.4%) | 8.761* | 0.067 | - | - | - |
|  | Moderate | 5 (41.7%) | 6 (75.0%) | 11 (29.7%) |  |  |  |  |  |
|  | Severe | 5 (41.7%) | 1 (12.5%) | 24 (64.9%) |  |  |  |  |  |
| Mayo score in ulcerative colitis | Median (IQR) | 8 (6 - 9.5) | 8 (7 - 9) | 11 (9 - 11) | 12.646≠ | 0.002 | 0.814 | 0.011 | 0.002 |
|  | Range | 4 – 12 | 5 – 9 | 4 – 12 |  |  |  |  |  |
| Mayo endoscopic scoring system | Median (IQR) | 2 (2 - 3) | 2 (2 - 2.5) | 3 (2 - 3) | 11.237≠ | 0.004 | 0.863 | 0.007 | 0.007 |
|  | Range | 1 – 3 | 1 – 3 | 1 – 3 |  |  |  |  |  |
| Location of disease in UC | Rectal | 1 (8.3%) | 2 (25.0%) | 2 (5.1%) | (3.987* | 0.408 | - | - | - |
|  | Left | 4 (33.3%) | 3 (37.5%) | 12 (30.8%) |  |  |  |  |  |
|  | Pancolitis | 7 (58.3%) | 3 (37.5%) | 25 (64.1%) |  |  |  |  |  |
|  | Pouchitis | 0 (0.0%) | 0 (0.0%) | 0 (0.0%) |  |  |  |  |  |
|  | Ileal affection | 0 (0.0%) | 0 (0.0%) | 0 0.0%) |  |  |  |  |  |
| Crohn's disease activity index | Median (IQR) | 191.5 (149 - 220) | 185 (171 - 259) | 187 (161 - 351) | 1.011≠ | 0.603 | - | - | - |
|  | Range | 134 – 267 | 161 – 329 | 139 – 452 |  |  |  |  |  |
| Simplified Crohn's activity index | Median (IQR) | 6 (5 - 6) | 5 (5 - 6) | 6 (5 - 7) | 1.699≠ | 0.428 | - | - | - |
|  | Range | 5 – 7 | 5 – 7 | 5 – 8 |  |  |  |  |  |
| Location of disease in Crohn's disease | Colonic | 1 (16.7%) | 0 0.0%) | 5 (23.8%) | 5.905* | 0.206 | - | - | - |
|  | Ileal | 1 (16.7%) | 0 (0.0%) | 8 (38.1%) |  |  |  |  |  |
|  | Ileocolonic | 4 (66.7%) | 4 (100.0%) | 8 (38.1%) |  |  |  |  |  |
|  | Small bowel | 0 (0.0%) | 0 (0.0%) | 0 (0.0%) |  |  |  |  |  |
|  | Gastric | 0 (0.0%) | 0 (0.0%) | 0 (0.0%) |  |  |  |  |  |
|  | Ileocecal & gastric | 0 (0.0%) | 0 (0.0%) | 0 (0.0%) |  |  |  |  |  |
| Dysplasia | No | 17 (94.4%) | 11 (91.7%) | 55 (91.7%) | 2.137* | 0.711 | - | - | - |
|  | Low grade | 1 (5.6%) | 0 (0.0%) | 3 (5.0%) |  |  |  |  |  |
|  | High-grade adenocarcinoma | 0 (0.0%) | 1 (8.3%) | 2 (3.3%) |  |  |  |  |  |
| Use of biological therapy | Yes | 12 (66.7%) | 6 (50.0%) | 24 (40.0%) | 4.018* | 0.134 | - | - | - |
|  | No | 6 (33.3%) | 6 (50.0%) | 36 (60.0%) |  |  |  |  |  |
| Type of used biological therapy | Infliximab | 6 (46.2%) | 2 (33.3%) | 9 (37.5%) | 6.138* | 0.804 | - | - | - |
|  | Adalilumab | 3 (23.1%) | 3 (50.0%) | 8 (33.3%) |  |  |  |  |  |
|  | Ustekinumab | 1 (7.7%) | 0 (0.0%) | 3 (12.5%) |  |  |  |  |  |
|  | Infliximab & ustekinumab | 2 (15.4%) | 0 (0.0%) | 2 (8.3%) |  |  |  |  |  |
|  | Infliximab & adalilumab | 1 (7.7%) | (0 (0.0%) | 1 (4.2%) |  |  |  |  |  |
|  | Adalilumab & ustekinumab | 0 0.0%) | 1 (16.7%) | 1 (4.2%) |  |  |  |  |  |
| Time from starting first biologics (years) | Mean±SD | 5.31 ± 3.54 | 2.67 ± 0.82 | 3.27 ± 1.88 | 3.733• | 0.033 | 0.033 | 0.019 | 0.588 |
|  | Range | 1.00 – 13.00 | 2.00 – 4.00 | 0.50 – 7.00 |  |  |  |  |  |
| Cause of using biological therapy | Steroid dependant | 6 (46.2%) | 2 (33.3%) | 11 (47.8%) | 4.372* | 0.626 | - | - | - |
|  | Steroid resistant | 4 (30.8%) | 2 (33.3%) | 9 (39.1%) |  |  |  |  |  |
|  | Extraintestinal | 1 (7.7%) | 1 (16.7%) | 3 (13.0%) |  |  |  |  |  |
|  | Fistula | 2 (15.4%) | 1 (16.7%) | 0 (0.0%) |  |  |  |  |  |

*: Chi-square test; •: One Way ANOVA test; ≠: Kruskall-Wallis test

P1: Comparison between 1^st^ degree and 2^nd^ degree

P2: Comparison between 1^st^ degree and group B

P3: Comparison between 2^nd^ degree and group B
