# Supplementary material for: Clinical characteristics of familial and sporadic inflammatory bowel disease in Egyptian patients
Source: BMC Gastroenterol. 2025 Dec 15;26:43. doi: 10.1186/s12876-025-04492-9 (PMC12821981; doi:10.1186/s12876-025-04492-9)
Supplement: Supplementary file 2 — Supplementary Material 2. Table S2. Subgroup outcomes: clinical response, remission and time-to-biologic in first-degree, second-degree and sporadic cases [file 12876_2025_4492_MOESM2_ESM.docx]

**Supplementary Table S2:** Comparison between 1^st^ degree relatives, 2^nd^ degree relatives and group B regarding the outcomes of the studied patients after 1 year

|  | | **1st degree** | **2nd degree** | **Group B** | **Test value** | **P-value** | **P1** | **P2** | **P3** |
| --- | --- | --- | --- | --- | --- | --- | --- | --- | --- |
|  |  | **No. = 18** | **No. = 12** | **No. = 60** |  |  |  |  |  |
| Mucosal healing at 1 year | Yes | 15 (83.3%) | 7 (58.3%) | 33 (55.0%) | 4.722 | 0.094 | - | - | - |
|  | No | 3 (16.7%) | 5 (41.7%) | 27 (45.0%) |  |  |  |  |  |
| Clinical response at 1 year | Yes | 16 (88.9%) | 9 (75.0%) | 31 (51.7%) | 9.123 | 0.010 | 0.317 | 0.005 | 0.137 |
|  | No | 2 (11.1%) | 3 (25.0%) | 29 (48.3%) |  |  |  |  |  |
| Clinical remission at 1 year | Yes | 16 (88.9%) | 9 (75.0%) | 31 (51.7%) | 9.123 | 0.010 | 0.317 | 0.005 | 0.137 |
|  | No | 2 (11.1%) | 3 (25.0%) | 29 (48.3%) |  |  |  |  |  |
| Normalisation of CRP at 1 year | Yes | 17 (94.4%) | 7 (58.3%) | 30 (50.0%) | 11.412 | 0.003 | 0.015 | 0.001 | 0.598 |
|  | No | 1 (5.6%) | 5 (41.7%) | 30 (50.0%) |  |  |  |  |  |
| Decrease in calprotectin at 1 year | Yes | 18 (100.0%) | 9 (75.0%) | 42 (70.0%) | 6.988 | 0.030 | 0.025 | 0.008 | 0.727 |
|  | No | 0 (0.0%) | 3 (25.0%) | 18 (30.0%) |  |  |  |  |  |

*: Chi-square test

P1: Comparison between 1^st^ degree and 2^nd^ degree

P2: Comparison between 1^st^ degree and group B

P3: Comparison between 2^nd^ degree and group B
